# Evaluation of left ventricular regional myocardial function by layer-specific strain in female patients with hypothyroidism

**DOI:** 10.3389/fcvm.2025.1489979

**Published:** 2025-05-23

**Authors:** Xiao Ding, Xijun Zhang, Changhua Wei, Jianjun Yuan, Kaikai Shen, Yisa Wang, Haohui Zhu

**Affiliations:** Department of Ultrasonography, Henan Provincial People’s Hospital, Zhengzhou Henan, China

**Keywords:** strain, hypothyroidism, systolic function, echocardiography, left ventricular

## Abstract

**Objective:**

This study aims to explore the value of layer-specific strain in evaluating the differences in left ventricular 18-segment myocardial function between female hypothyroidism patients and healthy controls.

**Methods:**

Thirty-two female hypothyroidism patients (hypothyroidism group, HG) with normal left ventricular global systolic function and 30 healthy female volunteers (control group, CG) underwent two-dimensional echocardiography. Offline analysis using EchoPAC113 software measured peak systolic circumferential strain (PCS) and peak systolic longitudinal strain (PLS). Layer-specific strain values (endocardium, mid-myocardium, epicardium) were compared between groups.

**Results:**

PLS of the endocardium (PLS-endo) of the posterior wall in the middle segments of the left ventricle in HG was lower than that in CG. PCS of the mid-myocardium (PCS-mid) and epicardium (PCS-epi) of all walls in the apical segments of the left ventricle in HG were lower than those in CG. In the middle segments of the left ventricle in HG, the PCS-epi of the anterior septum, PCS-mid and PCS-epi of the anterior wall, PCS-mid and PCS-epi of the inferior wall, and PCS-endo, PCS-mid, and PCS-epi of the lateral and posterior walls were lower than those in CG. In the basal segments of the left ventricle in HG, the PCS-mid and PCS-epi of the anterior wall, PCS-mid and PCS-epi of the posterior wall, PCS-epi of the inferior wall, and PCS-endo, PCS-mid, and PCS-epi of the lateral wall were lower than those in CG. The global circumferential strain (GCS) of the mid-myocardium and epicardium in the basal and apical segments, as well as all layers in the middle segment, was significantly lower in HG.

**Conclusions:**

The layer-specific strain values in the left ventricle in female patients with hypothyroidism differed from those of healthy female controls. When evaluating the left ventricular regional myocardial function in female patients with hypothyroidism, circumferential strain was, to a certain extent, more affected by thyroid hormone abnormalities than longitudinal strain.

## 1 Introduction

According to the Guidelines and Standards of the American Society of Echocardiography (ASE) and the European Association of Cardiovascular Imaging (EACVI) in 2015 (1), the left ventricle (LV) is divided into basal, middle, and apical segments, with each segment containing six walls (anterior septum, anterior wall, lateral wall, posterior wall, inferior wall, posterior septum), a total of 18 segments. LV myocardial fibers are arranged in a unique three-layer spiral pattern, with longitudinal myocardium in the endocardium, annular myocardium in the mid-myocardium, and oblique myocardium in the epicardium.

Left ventricular contraction consists of ventricular shortening and ventricular cavity shrinking. Ventricular shortening mainly involves the longitudinal systole of the endo- and epi-myocardium, while ventricular cavity shrinking is due to the circumferential systole of the mid-myocardium. Thus, the myocardial function of each layer in different LV segments varies and is differentially affected by various pathological factors. When single-layer or regional myocardial injury occurs, early changes in LV function can be observed, yet global systolic function may remain unchanged. Traditional ultrasound methods cannot analyze layer-specific strain in each LV segment, making it difficult to detect early myocardial function changes and potentially delaying diagnosis and treatment (2). In contrast, layer-specific strain, based on two-dimensional speckle-tracking imaging (STI) (3–9), can effectively analyze early or regional changes in LV myocardial function (10–14).

Hypothyroidism, a common clinical endocrine disorder, disproportionately affects women and is associated with cardiovascular complications (15–19). Previous studies (20, 21) showed that 4%–7% of the community-derived populations in the USA and Europe had undiagnosed hypothyroidism, and the incidence of clinical hypothyroidism in the United States was 1 in 300. The heart, highly sensitive to thyroid hormone (TH) levels, exhibits impaired contractility and relaxation in hypothyroidism, often preceding overt cardiac dysfunction. Previous research (15) indicated that 70%–80% of hypothyroidism patients had cardiovascular disease, and hypothyroidism was often associated with impaired LV systolic function (22–25). Sunbul et al. (17) reported that LV regional systolic function had been impaired in patients with hypothyroidism when global systolic function was normal. Biondi et al. (26) reported that a wide spectrum of cardiac abnormalities had long been recognized in patients with overt thyroid dysfunction. Moreover, insufficient thyroid hormone can lead to abnormal cardiac structure and weakened myocardial contraction and relaxation. If left untreated, it may progress to cardiac insufficiency (15). Early detection and treatment could improve the prognosis for most patients, highlighting the importance of early diagnosis of cardiac function changes in hypothyroidism patients (27).

Traditional ultrasonic inspection mainly focuses on LV global systolic and diastolic function. However, it is challenging to detect early LV systolic function changes and regional myocardial damage in patients with hypothyroidism using these methods, which may cause diagnostic delays (28–30). Layer-specific strain, by performing speckle-tracking analysis on the endocardium, mid-myocardium, and epicardium of LV 18 segments, can obtain two key parameters: peak systolic circumferential strain (PCS) and peak systolic longitudinal strain (PLS), along with their strain curves for each layer and the overall layer in corresponding LV segments, and display the relevant bull's-eye figure. These parameters can directly reflect the myocardial contractile function in different directions and layers, providing crucial information for evaluating regional myocardial function changes.

In this study, we aim to precisely quantify and compare PCS and PLS to explore the value of layer-specific strain in evaluating the differences in 18-segment LV myocardial function between female hypothyroidism patients and healthy controls. Additionally, we seek to determine which strain (CS or LS) is more sensitive to thyroid hormone imbalances, which could be key to enabling timely and effective clinical interventions.

## 2 Materials and methods

### 2.1 Study subjects

This study included female patients diagnosed with hypothyroidism who came to Henan Provincial People's Hospital from August 2018 to June 2019. The inclusion criteria were as follows: (1) The diagnosis of hypothyroidism, which included both subclinical and clinical hypothyroidism, was established in accordance with the clinical practice guidelines for hypothyroidism in adults jointly issued by the American Association of Clinical Endocrinologists and the American Thyroid Association in 2012 (31). (2) Conventional echocardiography showed no abnormalities in left ventricular configuration and function or only mild diastolic dysfunction. The exclusion criteria encompassed pregnant women; patients with hypothyroidism following hyperthyroidism; those with hypertension, diabetes, coronary heart disease, arrhythmia, valvular heart disease, myocarditis, or fibrosis (assessed by MRI); use of concomitant chemotherapy; patients with ischemic disease, possible unknown infiltrative disease, and other hypothyroidism with concurrent diseases influencing cardiac function. A total of 32 female patients [left ventricular ejection fraction (LVEF) ≥ 50%] with an average age of 40.7 ± 10.8 years and an average disease duration of 4.6 ± 0.7 years were selected. Additionally, 30 age-matched female healthy volunteers were randomly recruited as the control group (CG), with an average age of 41.0 ± 13.1 years and without cardiovascular diseases or other vital organ diseases, as confirmed by physical examination, electrocardiogram (ECG), and echocardiography. This study was approved by the Ethics Committee of Henan Provincial People's Hospital (Zhengzhou, Henan, China) with a serial number of 2019 and an Ethical Review No. 34. Informed consent was obtained from all participants.

All the patients included in this study were females. This selection was not arbitrary. It is well established in the medical literature that the prevalence of hypothyroidism is significantly higher in women compared with men (19–21). By focusing on female patients, we aimed to create a more homogeneous study population, which would enhance the detectability of the potential effects of hypothyroidism on left ventricular myocardial function. This approach allowed us to reduce the confounding factors associated with gender differences in cardiac physiology and hormonal regulation. In future research, we plan to expand our investigation to include male patients as well, to comprehensively assess the impact of hypothyroidism on cardiac function across genders.

### 2.2 Echocardiography and instruments

The GE Vivid E9 color Doppler ultrasound diagnostic apparatus, equipped with a two-dimensional probe M5S (frequency, 2.0–4.5 MHz; frame rate, 50–70 frames/s), was utilized. Subjects were positioned in the left lateral decubitus position and instructed to breathe calmly. A chest lead ECG was recorded synchronously.

Conventional echocardiography was employed to measure the left ventricular end-systolic diameter (LVIDs), left ventricular end-diastolic diameter (LVIDd), interventricular septum end-diastolic thickness (IVSD), and left ventricular posterior wall end-diastolic thickness (LVPWD). The left ventricular ejection fraction (LVEF) was measured in the LV apical four-chamber and apical two-chamber views using the biplane Simpson's method. All measurements were repeated three times by two experienced clinicians to obtain the mean values. For image acquisition, only images with excellent quality, including clear myocardial borders, good contrast, and stable motion, were selected. Two-dimensional grayscale dynamic images of the apical LV long-axis view, four-chamber view, two-chamber view, and left ventricular short-axis views at the mitral valve, papillary muscle, and apical levels were collected for three consecutive cardiac cycles and stored on a CD.

**Figure 1:**
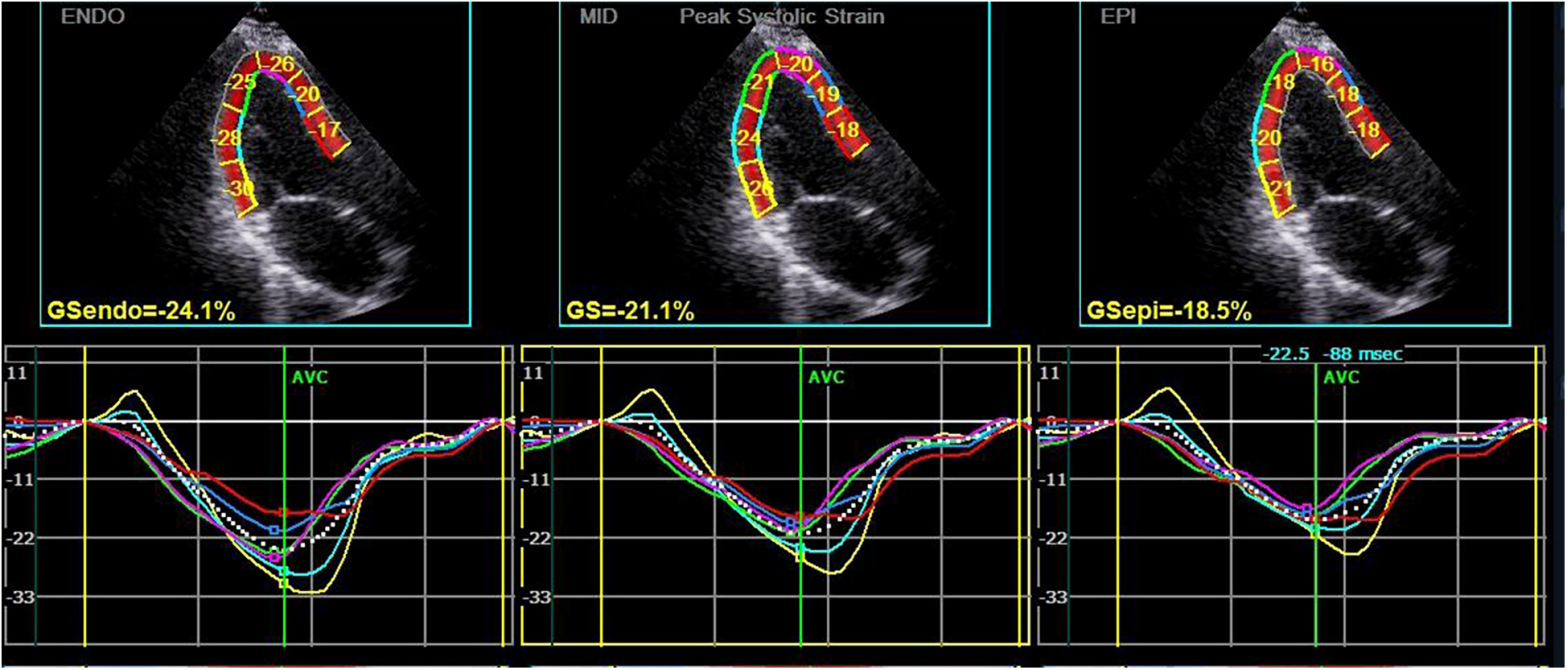
Longitudinal strain and strain curve of the left ventricular myocardium in a hypothyroidism patient. The upper half of the picture depicts the peak systolic longitudinal strain of the endocardium, mid-myocardium, and epicardium (PLS-endo, PLS-mid, and PLS-epi) values of each segment in apical left ventricular long-axis view, and the lower half shows the strain curve corresponding to each segment.

**Figure 2:**
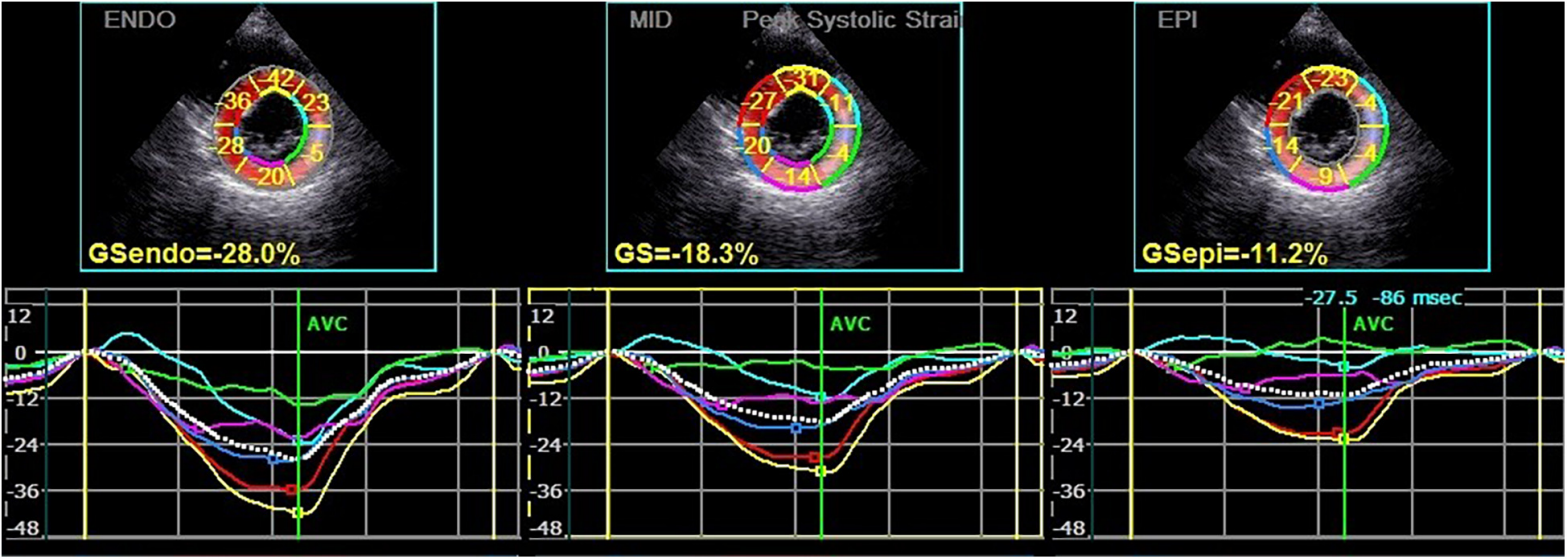
Circumferential strain and strain curve of the left ventricular myocardium in a hypothyroidism patient. The upper half of the picture depicts the peak systolic circumferential strain of the endocardium, mid-myocardium, and epicardium (PCS-endo, PCS-mid, and PCS-epi) values of each segment in left ventricular short-axis mitral valve level view, and the lower half shows the strain curve corresponding to each segment.

Subsequently, the stored dynamic images were Q-analyzed for 2D strain using the offline EchoPAC113 software. The endocardium of the dynamic images from six views (short-axis and long-axis of the left ventricular) was manually traced. After that, the system automatically segmented the left ventricular wall into inner, middle, and outer myocardial layers, and speckle-tracking analysis was carried out on each layer. Manual adjustment of the region of interest was permitted to ensure optimal tracking quality. The system divided each LV base segment, middle segment, and apical segment into six walls (anterior septum, anterior wall, lateral wall, posterior wall, inferior wall, and posterior septum), resulting in a total of 18 segments (1). Peak longitudinal strain (PLS), peak circumferential strain (PCS), global longitudinal strain (GLS), global circumferential strain (GCS), and the corresponding strain curves for each cardiac cycle were automatically generated (Figures 1, 2), and the relevant bull's-eye figure diagrams were displayed.

To minimize potential bias in the echocardiographic analysis, we implemented a blinding method. The operators who performed the speckle-tracking analysis using the EchoPAC113 software were blinded to the group assignment of the patients [hypothyroidism group (HG) or control group]. This was achieved by removing any patient-identifying information related to the diagnosis from the images before analysis, ensuring that the interpretation of the myocardial strain parameters was not influenced by prior knowledge of the patient's condition.

During the image acquisition process, we evaluated the quality of the images. Images were considered suboptimal if they had unclear myocardial borders, poor contrast, or unstable motion. For these cases, we made efforts to adjust the acquisition parameters or reacquire the images.

### 2.3 Statistical analysis

SPSS 17.0 software was selected for statistical analysis. Continuous variables were expressed as mean ± SD. The normality of the studied parameters was tested using the Shapiro–Wilk test. For normally distributed data, an independent-samples *t* test was used for comparison. For data that did not follow a normal distribution, non-parametric tests were applied. The intra- and inter-class correlation coefficients (ICCs) were calculated to assess the repeatability of the measurements, and inter- and intra-observer repeatability tests were carried out. *P* < 0.05 was considered statistically significant. In the context of multiple testing, the Sidak correction method was implemented to adjust the significance levels. The Sidak correction is calculated as 1 −  (1 − *α*)^1/*m*^, where *α* is the original significance level and *m* is the number of tests. By doing so, we effectively controlled the type I error rate under the condition of multiple tests, thereby ensuring the robustness and reliability of our statistical inferences.

## 3 Results

### 3.1 General clinical data and 2D echocardiographic parameters of included participants

The general clinical data parameters in HG and CG are presented in Table 1. There were no significant differences in average age, height, weight, body mass index (BMI), heart rate (HR), systolic blood pressure (SBP), and diastolic blood pressure (DBP) between HG and CG (all *P* > 0.05). The evaluation of the 2D echocardiographic measurements in HG and CG is summarized in Table 2. There were also no significant differences in LVIDd, LVIDs, IVSD, LVPWD, and LVEF between the two groups (all *P* > 0.05).

**Table 1:** Comparison of general clinical data parameters in HG and CG^a^.

Group	Numbers	Age (years)	Height (cm)	Weigh (kg)	BMI (kg/m^2^)	HR (beats/min)	SBP (mmHg)	DBP (mmHg)
CG	30	41.0 ± 13.1	162.3 ± 4.8	57.1 ± 5.9	20.5 ± 2.4	66.3 ± 9.6	119.45 ± 8.45	72.77 ± 6.88
HG	32	40.7 ± 10.8	161.1 ± 5.8	56.7 ± 6.1	22.1 ± 2.1	65.3 ± 9.5	118.96 ± 9.01	69.99 ± 7.82
*T-*value		0.230	−1.161	−0.972	−1.461	0.712	0.485	1.175
*P-*value		0.821	0.244	0.304	0.170	0.421	0.791	0.313

^a^ Data are presented as mean ± SD.

CG, control group; HG, hypothyroidism group; BMI, body mass index; HR, heart rate; SBP, systolic blood pressure; DBP, diastolic blood pressure.

**Table 2:** Comparison of conventional echocardiographic parameters in HG and CG^a^.

Group	Numbers	LVEF (%)	LVIDd (mm)	LVIDs (mm)	IVSD (mm)	LVPWD (mm)
CG	30	65.2 ± 3.8	46.3 ± 2.2	31.3 ± 2.9	7.8 ± 1.0	7.8 ± 0.7
HG	32	64.5 ± 4.4	48.3 ± 2.6	31.2 ± 2.4	8.0 ± 1.0	8.2 ± 1.1
*T*-value		−0.401	1.032	0.174	0.333	0.591
*P*-value		0.722	0.091	0.891	0.623	0.370

^a^ Data are presented as mean ± SD.

CG, control group; HG, hypothyroidism group; LVEF, left ventricular ejection fraction; LVIDd, left ventricular end-diastole diameter; LViDs, left ventricular end-systolic diameter; IVSD, interventricular septum end-diastolic thickness; LVPWD, left ventricular posterior wall end-diastolic thickness.

### 3.2 Comparison of myocardial layer-specific strain parameters

Longitudinal strain (LS): Table 3 presents the layer-specific longitudinal strain of the posterior wall in the middle LV segment for HG and CG. In HG, the peak systolic longitudinal strain of the endocardium (PLS-endo) of the posterior wall in the middle LV segments was lower than that in CG (*P* < 0.05). However, there were no significant differences in the peak systolic longitudinal strain of the mid-myocardium (PLS-mid) and epicardium (PLS-epi) of the posterior wall in the middle LV segments between the two groups (both *P* > 0.05). Similarly, no significant differences were observed in the PLS of other LV myocardial segments (*P* > 0.05).

**Table 3:** Comparison of layer-specific longitudinal strain of the posterior wall in the left ventricular middle segment in HG and CG^a^.

Group	midpostPLSEndo (%)	midpostPLSMid (%)	midpostPLSEpi (%)
HG	−23.31 ± 3.56	−21.69 ± 3.23	−20.19 ± 3.14
CG	−25.84 ± 1.70	−23.08 ± 2.04	−20.70 ± 2.44
*T*-value	−2.161	−1.130	−4.000
*P*-value	0.046	0.273	0.694

^a^ Data are presented as mean ± SD.

CG, control group; HG, hypothyroidism group; midpostPLSEndo, peak longitudinal strain in the inner layer myocardium of the posterior wall in the middle left ventricular segment; midpostPLSMid, peak longitudinal strain in the middle layer myocardium of the posterior wall in the middle left ventricular segment; midpostPLSEpi, peak longitudinal strain in the outer layer myocardium of the posterior wall in the middle left ventricular segment.

Circumferential strain (CS): Table 4 presents the layer-specific circumferential strains of each wall in the apical LV segment for HG and CG. In the apex LV segments of HG, the peak systolic circumferential strains of the mid-myocardium (PCS-mid) and epicardium (PCS-epi) of all walls were lower than those in CG (all *P* < 0.05), whereas the differences in the peak systolic circumferential strains of the endocardium (PCS-endo) of all walls were not significant (*P* > 0.05). Table 5 shows the layer-specific circumferential strains of each wall in the middle LV segment for the two groups. In the middle LV segments of HG, compared with CG, the PCS-endo of most walls (except the posterior septum) was lower. The PCS-endo of the lateral and posterior walls exhibited significant differences (*P* = 0.000), while no significant differences were detected in those of the anterior septum, anterior wall, and inferior wall (all *P* > 0.05). The PCS-mid of the anterior wall, PCS-mid of the lateral wall, PCS-mid of the posterior wall, and PCS-mid of the inferior wall in HG were significantly lower than those in CG, while the differences in those of the anterior septum and posterior septum were not significant (both *P* > 0.05). The PCS-epi of the anterior septum, PCS-epi of the anterior wall, PCS-epi of the lateral wall, PCS-epi of the posterior wall, and PCS-epi of the inferior wall in HG were also significantly lower, with the difference in PCS-epi of the posterior septum being not significant (*P* > 0.05). Table 6 presents the layer-specific circumferential strains of each wall in the basal LV segment for HG and CG. In the basal LV segments of HG, the PCS-mid of the anterior wall, PCS-mid of the lateral wall, and PCS-mid of the posterior wall were lower, while the differences in those of the anterior septum, inferior wall, and posterior septum were not significant (all *P* > 0.05). The PCS-epi of the anterior wall, PCS-epi of the lateral wall, PCS-epi of the posterior wall, and PCS-epi of the inferior wall were lower, while the differences in PCS-epi of the anterior septum and posterior septum were not significant (both *P* > 0.05). The PCS-endo of the lateral wall was lower, while there were no significant differences in other LV myocardial segments (*P* > 0.05).

**Table 4:** Comparison of layer-specific circumferential strain of each wall in the left ventricular apical segment in HG and CG^a^.

Group		HG	CG	*P*-value
antseptPCS (%)	Endo	−34.94 ± 20.83	−43.66 ± 12.98	0.284
	Mid	−22.69 ± 13.38	−33.77 ± 8.52	0.043
	Epi	−14.59 ± 8.97	−27.08 ± 6.13	0.002
antPCS (%)	Endo	−33.74 ± 21.18	−43.61 ± 12.22	0.228
	Mid	−21.36 ± 14.45	−33.61 ± 8.66	0.037
	Epi	−13.61 ± 10.15	−26.94 ± 7.07	0.003
latPCS (%)	Endo	−32.72 ± 19.62	−44.26 ± 10.69	0.129
	Mid	−19.99 ± 13.03	−34.30 ± 6.66	0.007
	Epi	−12.22 ± 8.98	−27.48 ± 4.98	0.000
postPCS (%)	Endo	−35.51 ± 16.95	−43.97 ± 10.97	0.208
	Mid	−23.37 ± 9.59	−34.21 ± 6.75	0.009
	Epi	−15.34 ± 6.74	−27.58 ± 4.81	0.000
infPCS (%)	Endo	−39.57 ± 13.74	−44.17 ± 11.83	0.431
	Mid	−27.37 ± 7.00	−34.35 ± 7.73	0.043
	Epi	−19.23 ± 5.18	−27.61 ± 5.91	0.003
postseptPCS (%)	Endo	−38.84 ± 15.11	−44.28 ± 13.05	0.398
	Mid	−25.51 ± 9.67	−34.31 ± 9.15	0.049
	Epi	−16.46 ± 7.79	−27.46 ± 7.49	0.004

^a^ Data are presented as mean ± SD.

CG, control group; HG, hypothyroidism group; Endo, in the inner layer myocardium; Mid, in the middle layer myocardium; Epi, in the outer layer myocardium; antseptPCS, peak circumferential strain of the anterior septum; antPCS, peak circumferential strain of the anterior wall; latPCS, peak circumferential strain of the lateral wall; postPCS, peak circumferential strain of the posterior wall; infPCS, peak circumferential strain of the inferior wall; postseptPCS, peak circumferential strain of the posterior septum.

**Table 5:** Comparison of layer-specific circumferential strain of each wall in the left ventricular middle segment in HG and CG^a^.

Group		HG	CG	*P*-value
antseptPCS (%)	Endo	−36.15 ± 7.31	−36.71 ± 5.89	0.850
	Mid	−24.60 ± 6.48	−28.76 ± 3.99	0.107
	Epi	−15.88 ± 6.30	−22.82 ± 2.98	0.007
antPCS (%)	Endo	−24.20 ± 12.83	−32.94 ± 6.70	0.080
	Mid	−15.26 ± 8.79	−25.55 ± 4.73	0.005
	Epi	−8.59 ± 6.30	−20.30 ± 3.92	0.000
latPCS (%)	Endo	−15.39 ± 11.57	−32.92 ± 5.38	0.000
	Mid	−7.94 ± 8.32	−25.14 ± 4.35	0.000
	Epi	−3.30 ± 6.50	−19.55 ± 4.65	0.000
postPCS (%)	Endo	−21.97 ± 7.23	−35.88 ± 4.85	0.000
	Mid	−12.21 ± 5.95	−28.04 ± 3.48	0.000
	Epi	−6.67 ± 4.20	−22.25 ± 3.27	0.000
infPCS (%)	Endo	−33.69 ± 7.99	−36.88 ± 5.82	0.324
	Mid	−22.82 ± 7.01	−30.17 ± 4.61	0.013
	Epi	−14.60 ± 6.73	−25.01 ± 3.74	0.001
postseptPCS (%)	Endo	−39.87 ± 6.16	−38.16 ± 5.57	0.520
	Mid	−28.66 ± 6.08	−30.62 ± 4.87	0.437
	Epi	−20.09 ± 6.23	−24.96 ± 4.64	0.064

^a^ Data are presented as mean ± SD.

CG, control group; HG, hypothyroidism group; Endo, in the inner layer myocardium; Mid, in the middle layer myocardium; Epi, in the outer layer myocardium; antseptPCS, peak circumferential strain of the anterior septum; antPCS, peak circumferential strain of the anterior wall; latPCS, peak circumferential strain of the lateral wall; postPCS, peak circumferential strain of the posterior wall; infPCS, peak circumferential strain of the inferior wall; postseptPCS, peak circumferential strain of the posterior septum.

**Table 6:** Comparison of layer-specific circumferential strain of each wall in the left ventricular basal segment in HG and CG^a^.

Group		HG	CG	*P*-value
antseptPCS (%)	Endo	−39.33 ± 11.15	−36.05 ± 5.84	0.434
	Mid	−28.99 ± 10.14	−29.35 ± 4.89	0.924
	Epi	−21.71 ± 8.05	−24.23 ± 4.45	0.410
antPCS (%)	Endo	−25.56 ± 10.72	−29.03 ± 6.15	0.397
	Mid	−16.78 ± 8.29	−23.94 ± 4.22	0.029
	Epi	−10.49 ± 7.65	−20.31 ± 3.21	0.002
latPCS (%)	Endo	−16.34 ± 10.66	−24.75 ± 5.53	0.031
	Mid	−9.92 ± 7.61	−20.67 ± 4.37	0.001
	Epi	−6.02 ± 6.71	−17.61 ± 3.58	0.000
postPCS (%)	Endo	−20.66 ± 11.58	−25.55 ± 5.05	0.210
	Mid	−13.53 ± 7.55	−19.76 ± 3.76	0.035
	Epi	−8.39 ± 5.81	−15.61 ± 2.73	0.002
infPCS (%)	Endo	−30.66 ± 10.73	−32.28 ± 5.07	0.680
	Mid	−21.05 ± 7.16	−25.31 ± 4.31	0.132
	Epi	−13.89 ± 5.43	−19.96 ± 3.79	0.010
postseptPCS (%)	Endo	−38.94 ± 7.19	−37.68 ± 8.17	0.714
	Mid	−29.56 ± 6.97	−30.92 ± 6.55	0.655
	Epi	−22.33 ± 6.81	−25.63 ± 5.32	0.245

^a^ Data are presented as mean ± SD.

CG, control group; HG, hypothyroidism group; Endo, in the inner layer myocardium; Mid, in the middle layer myocardium; Epi, in the outer layer myocardium; antseptPCS, peak circumferential strain of the anterior septum; antPCS, peak circumferential strain of the anterior wall; latPCS, peak circumferential strain of the lateral wall; postPCS, peak circumferential strain of the posterior wall; infPCS, peak circumferential strain of the inferior wall; postseptPCS, peak circumferential strain of the posterior septum.

### 3.3 Comparison of GLS and GCS parameters

Table 7 summarizes the results of the evaluation of GLS in HG and CG. The values of global longitudinal strain of the endocardium (GLS-endo), global longitudinal strain of the mid-myocardium (GLS-mid), global longitudinal strain of the epicardium (GLS-epi), and overall GLS were lower in HG than those in CG, but the differences were not statistically significant (*P* > 0.05). The results of the evaluation of GCS in the LV basal, middle, and apical segments in HG and CG are shown in Table 8. In the LV basal and apical segments in HG, the values of global circumferential strain of the mid-myocardium (GCSMid) and epicardium (GCSEpi) were significantly lower than those in CG (*P* < 0.05). In the LV middle segment, the values of global circumferential strain of the endocardium (GCSEndo), GCSMid, and GCSEpi were significantly lower (*P* < 0.05). Nevertheless, there were no significant differences in GCSEndo in the LV basal and apical segments between the two groups (*P* > 0.05).

**Table 7:** Comparison of GLS in the left ventricle in HG and CG^a^.

Group	GLS-endo	GLS-mid	GLS-epi	GLS
CG	−25.24 ± 2.55	−22.33 ± 2.36	−20.10 ± 1.79	−23.58 ± 2.27
HG	−25.06 ± 1.55	−21.99 ± 1.47	−19.38 ± 1.47	−22.19 ± 1.59
*T*-value	−0.194	−0.383	−1.019	−0.379
*P*-value	0.849	0.708	0.321	0.654

^a^ Data are presented as mean ± SD.

CG, control group; HG, hypothyroidism group; GLS-endo, global longitudinal strain in the left ventricular inner layer myocardium; GLS-mid, global longitudinal strain in the left ventricular middle layer myocardium; GLS-epi, global longitudinal strain in the left ventricular outer layer myocardium; GLS, global longitudinal strain.

**Table 8:** Comparison of GCS in the left ventricular basal, middle, and apical segments in HG and CG^a^.

Group	bGCSEndo	bGCSMid	bGCSEpi
CG	−28.84 ± 4.55	−19.12 ± 3.86	−12.40 ± 4.56
HG	−31.33 ± 4.06	−25.25 ± 3.40	−20.61 ± 2.98
*P*-value	0.210	0.001	0.000
Group	mGCSEndo	mGCSMid	mGCSEpi
CG	−29.70 ± 3.32	−18.18 ± 3.41	−10.03 ± 3.49
HG	−35.45 ± 4.56	−27.95 ± 2.99	−22.43 ± 2.45
*P*-value	0.003	0.000	0.000
Group	aGCSEndo	aGCSMid	aGCSEpi
CG	−36.47 ± 16.52	−23.63 ± 9.33	−15.32 ± 6.25
HG	−44.27 ± 11.44	−34.23 ± 7.28	−27.51 ± 5.22
*P*-value	0.241	0.011	0.000

^a^ Data are presented as mean ± SD.

CG, control group; HG, hypothyroidism group; bGCSEndo, global circumferential strain in the inner layer myocardium of the left ventricular basal segment; bGCSMid, global circumferential strain in the middle layer myocardium of the left ventricular basal segment; bGCSEpi, global circumferential strain in the outer layer myocardium of the left ventricular basal segment; mGCSEndo, global circumferential strain in the inner layer myocardium of the left ventricular middle segment; mGCSMid, global circumferential strain in the middle layer myocardium of the left ventricular middle segment; mGCSEpi, global circumferential strain in the outer layer myocardium of the left ventricular middle segment; aGCSEndo, global circumferential strain in the inner layer myocardium of the left ventricular apical segment; aGCSMid, global circumferential strain in the middle layer myocardium of the left ventricular apical segment; aGCSEpi, global circumferential strain in the outer layer myocardium of the left ventricular apical segment.

### 3.4 Repeatability parameters

Fifteen patients were randomly selected to assess the inter- and intra-observer repeatability. The intra- and inter-observer intraclass correlation coefficient (ICC) values for the PCS-mid of the posterior wall were 0.959 and 0.933, respectively. The ICC values were 0.981 and 0.941 for the inferior wall, 0.967 and 0.923 for the anterior septum in the middle LV segment, 0.971 and 0.943 for the anterior wall, 0.954 and 0.969 for the posterior septum, and 0.977 and 0.931 for the lateral wall. These results indicated good repeatability.

During the data collection process of this study, due to the integration difficulties of patient data from multiple referral medical institutions and the initial focus on myocardial strain-related indicators, the thyroid function and current treatment data of HG patients could not be systematically collected. This is a shortcoming of this study, and it will be improved in subsequent research.

## 4 Discussion

This study investigated the myocardial strain parameters in HG compared with CG, revealing distinct patterns of left ventricular dysfunction. Key findings and their implications are discussed below. Longitudinal strain findings: In the middle LV segments, the PLS-endo, PLS-mid, and PLS-epi of the posterior wall in HG were lower than those in CG. However, only PLS-endo reached statistical significance. This suggests preferential vulnerability of the endocardial layer to hypothyroidism-induced damage, consistent with its higher energy demand and contractile role in consistent with (14, 32). Tafarshiku et al. (32) similarly reported subendocardial impairment in hypothyroidism, likely reflecting microcirculation dysfunction. Those also cannot exclude the reduction of endocardial resolution or error caused by the LV posterior wall being located in the distant field in the acquisition of images. Notably, GLS parameters, including GLS-endo, GLS-mid, GLS-epi, and overall GLS, were lower in HG but not statistically significant. This indicated that the longitudinal function of the LV myocardium demonstrated trends of impairment in HG. However, the cumulative effect on global longitudinal function was not of sufficient magnitude to achieve statistical significance. The normal LVEF in HG, despite the varying degrees of change to the LV myocardium, is consistent with previous research (33). This may be due to the compensatory mechanisms of the heart, where the remaining healthy myocardial tissue can maintain overall cardiac output, masking the subclinical myocardial damage detected by strain analysis.

Circumferential strain findings: CS abnormalities were more pronounced. PCS-mid and PCS-epi of all walls in HG were significantly lower in the apical LV segments. Middle and basal LV segments showed layer-specific reductions, particularly in the lateral and posterior walls. GCSMid and GCSEpi in the basal and apical segments, and all layers in the middle segment in HG, were significantly impaired. These results clearly indicate that left ventricular regional circumferential motion was impaired in hypothyroidism patients, which is consistent with previous studies (32, 34). The differences in CS among the 18 LV segments in HG were not uniform. Left ventricular circumferential motion, which mainly contributes to ventricular cavity narrowing, is mainly driven by the mid- and epi-myocardium, with the endo-myocardium playing a relatively smaller role. When thyroid hormone is deficient, the middle and outer myocardia are more affected. Among the LV basal, middle, and apical segments, the middle segment plays a dominant role in LV circumferential motion. Therefore, the full-thickness myocardium in the middle segment is the first to show damage, which explains the more significant differences in CS parameters in the middle LV segments.

Previous studies (18, 27, 29) have reported several mechanisms underlying the impaired left ventricular myocardial function in patients with hypothyroidism. Firstly, thyroid hormone (TH) deficiency leads to an upregulation of the cardiac cell membrane β-receptor by triiodothyronine, an increase in sarcoplasmic reticulum phosphoprotein expression, and a decrease in sarcoplasmic reticulum calcium–adenosine triphosphatase activity. This sequence of events weakens the myocardial contraction and relaxation functions. Secondly, TH insufficiency causes an increase in capillary permeability, local accumulation of mucin and mucopolysaccharides, and tissue fluid leakage, resulting in myocardial mucinous edema, myofibrillary degeneration, and necrosis, ultimately leading to weakened myocardial contractility and diastolic function. Thirdly, the insufficient action of TH weakens the diastolic effect of triiodothyronine (T3) on vascular smooth muscle, increasing systemic vascular resistance and circulatory overload, which in turn reduces myocardial contractility, cardiac output, and the systolic and diastolic functions of the left ventricle.

Our study confirms that layer-specific circumferential strain can provide more useful references for evaluating left ventricular regional myocardial function in hypothyroidism patients compared with layer-specific longitudinal strain. This indicates that CS is more sensitive to the impact of hypothyroidism on the LV myocardium. Given the significant differences in left ventricular layer-specific strain values between female hypothyroidism patients and healthy female controls, layer-specific strain may serve as a novel indicator for early evaluation of cardiac function changes in hypothyroidism patients. While layer-specific strain has been studied in cardiac function assessment in general, its application in specifically detecting early cardiac function changes in hypothyroidism represents a new area of exploration. Subsequent longitudinal studies can be carried out to longitudinally follow up patients with hypothyroidism and dynamically monitor changes in myocardial strain, to more accurately reveal the development order and mechanism of myocardial damage.

During the design phase of this study, the potential influence of disease duration on myocardial function was not comprehensively taken into account. Moreover, this crucial information was not systematically documented during the data collection process. This oversight constitutes one of the limitations of the present study. Numerous prior studies (23, 27, 29) have demonstrated that as thyroid-stimulating hormone (TSH) levels increase or free triiodothyronine (FT3) levels decrease, the impairment of left ventricular function tends to exacerbate. Additionally, the longer the myocardium remains in a state of thyroid hormone deficiency, the more likely it is to gradually intensify myocardial cell metabolic disorders, thereby exerting a more pronounced impact on myocardial function. In future research endeavors, it is imperative to incorporate the factor of disease duration for stratification analysis. This approach will enable a more in-depth clarification of its correlation with myocardial strain, potentially providing novel insights into the complex relationship between thyroid-related conditions and cardiac function.

Moreover, this study has certain limitations. It is a single-center study with a relatively small sample size, which may limit the generalizability of the results. A large-sample analysis of the correlation between thyroid hormone and myocardial strain is lacking. Additionally, the specific effects and roles of thyroid hormone levels on the endocardial, mid-myocardial, and epicardial layers remain unclear and require further research. Layer-specific strain, an important parameter in our study, is constrained by its reliance on spatial resolution and the high-standard requirements for image quality. Importantly, during the operation, we failed to record the number of segments that required manual adjustment or were excluded because of poor quality. Given that the quality of the analyzed segments is fundamental to the validity of the strain measurement results, the lack of this information may potentially undermine the reliability and accuracy of our findings.

In conclusion, future research should focus on three key areas. First, expand the sample size to boost statistical power and generalizability. Second, conduct multicenter research to reduce biases. Third, explore the underlying mechanisms connecting hypothyroidism to left ventricular myocardial function changes, especially at the cellular and molecular levels of different myocardial layers. Doing so can enhance the clinical value of layer-specific strain in evaluating left ventricular function in patients with hypothyroidism. This may enable earlier detection of cardiac complications, leading to more timely and effective treatment. Moreover, we’ll implement a comprehensive quality-control protocol for future studies to ensure high-standard data collection, image acquisition, and analysis, minimizing errors in measuring layer-specific strain.

## Data Availability

The raw data supporting the conclusions of this article will be made available by the authors, without undue reservation.
